# Tip110 interacts with YB-1 and regulates each other’s function

**DOI:** 10.1186/1471-2199-14-14

**Published:** 2013-07-04

**Authors:** Khalid Amine Timani, Ying Liu, Johnny J He

**Affiliations:** 1 University of North Texas Health Science Center, 3500 Camp Bowie Blvd., Fort Worth, TX 76107, USA

**Keywords:** HIV-1 Tat, Tip110, YB-1, Alternative Splicing, CD44, Transcription

## Abstract

**Background:**

Tip110 plays important roles in tumor immunobiology, pre-mRNA splicing, expression regulation of viral and host genes, and possibly protein turnover. It is clear that our understanding of Tip110 biological function remains incomplete.

**Results:**

Herein, we employed an immunoaffinity-based enrichment approach combined with protein mass spectrometry and attempted to identify Tip110-interacting cellular proteins. A total of 13 major proteins were identified to be complexed with Tip110. Among them was Y-box binding protein 1 (YB-1). The interaction of Tip110 with YB-1 was further dissected and confirmed to be specific and involve the N-terminal of both Tip110 and YB-1 proteins. A HIV-1 LTR promoter-driven reporter gene assay and a CD44 minigene *in vivo* splicing assay were chosen to evaluate the functional relevance of the Tip110/YB-1 interaction. We showed that YB-1 potentiates the Tip110/Tat-mediated transactivation of the HIV-1 LTR promoter while Tip110 promotes the inclusion of the exon 5 in CD44 minigene alternative splicing.

**Conclusions:**

Tip110 and YB-1 interact to form a complex and mutually regulate each other’s biological functions.

## Background

HIV-1 Tat-interacting protein of 110 kDa (Tip110), also known as squamous cell carcinoma antigen recognized by T cells 3 (SART3), was initially identified from a human myeloid cell line KG-1 cDNA library in 1995 [1]. Several important biological functions have been attributed to this gene/protein since its identification. A considerably elevated level of Tip110 is detected in a variety of human cancers [2-9], it has been proposed as a tumor antigen for immunotherapy. In addition, Tip110 binds to small nuclear RNA U6 and regulates eukaryotic pre-mRNA splicing ( [10,11] and our unpublished data). It also preferentially regulates the inclusion of exon 1a and skipping of exon 1b of the OCT4 gene [12]. Alteration of splicing components induced by a mutation in *early grey*, a Tip110 orthrologue in zebrafish leads to organ-specific defects and embryonic death [13], suggesting an important role of Tip110 in development. Furthermore, Tip110 is directly involved in expression regulation of viral and host genes including HIV-1, androgen receptor, and stem cell factors CYMC, GATA-2, NANOG, and SOX2 [14-17]. Lastly, Tip110 is shown to interact with ubiquitin-specific peptidases (USP) such as USP4 and regulate protein degradation [18]. It is clear that our understanding of Tip110 biological function is still rapidly evolving.

To further understand the biological function of Tip110, we wished to begin by identifying potential cellular proteins that interacted with Tip110. We took the immunoaffinity-based enrichment approach, followed by protein mass spectrometry. We isolated a total of 13 Tip110-binding proteins. Among them was Y-box binding protein 1 (YB-1). In this study, we characterized Tip110/YB-1 interaction and its impacts on each other’s function.

## Methods

**Cell culture and transfection** 293T cells were purchased from American Tissue Culture Collection (ATCC) and grown in Dulbecco’s modified Eagle’s medium containing 10% fetal bovine serum. The cell line was maintained in 100 IU/ml penicillin-100 μg/ml streptomycin and incubated at 37°C in 5% CO_2_. Cells were transfected using the standard calcium phosphate precipitation method. The chloramphenicol acetyltransferase (CAT) reporter gene assay was performed as described previously [14].

**Plasmids** Construction of pTip110-His, pTip110-HA, ΔCT, ΔRRM, ΔNT, ΔNLS, pTip110-GFP, pTat-Myc plasmids have been described elsewhere [14,15]. The pCD44 minigene plasmid was generously provided by Dr. Stefan Stamm of University of Kentucky, Lexington, KY). The full length YB-1 cDNA (1-324aa) and the deletion mutants pYB-1ΔC (1-128aa) and pYB-1ΔN (129-324aa) were generated by standard PCR techniques and inserted into the mammalian expression plasmid pCMV-Myc by using the pSPORT6-YB-1 plasmid as a template. Primers were for pYB-1-Myc: 5′-ATC CG*A GAT CT*A TAG CAG CGA GGC CGA GAC-3′ and 5′-AAT AC*C TCG AG*T TAC TCA GCC CCG CCC TGC TC-3′; for pYB-1ΔC: 5′-ATC CG*A GAT CT*A TAG CAG CGA GGC CGA GAC-3′ and 5′-AAT AC*C TCG AG*T TAA GGA CCT GTA ACA TTT GCT GC-3′; for pYB-1ΔN: 5′-ATC CG*A GAT CT*A TGG TGG TGT TCC AGT TCA AGG-3′ and 5′-AAT AC*C TCG AG*T TAC TCA GCC CCG CCC TGC TC-3′. Respective Bgl II and Xho I restriction sites in the primers were italicized.

**Mass spectrometry** 293T cells were mock or pTip110-HA transfected. At 72 hr post-transfection, the cells were lysed with WCEB buffer (50 mM Tris.HCl, pH 8.0, 280 mM NaCl, 0.5% NP-40, 0.2 mM EDTA, 2 mM EGTA, 10% glycerol, 2 mM PMSF and protease inhibitors) and applied to the anti-HA affinity matrix column (Roche). The column was washed with 20 bed-volumes, using washing buffer (20 mM Tris.HCl, pH 7.5, 0.1 M NaCl; 0.1 mM EDTA) and then incubated at 37°C with elution buffer containing HA peptide (1 mg/ml) for 15 min. A portion of the elutes was mixed with 4× SDS-PAGE loading buffer and then resolved by 10% SDS-PAGE, and the remaining elutions were analyzed by LC-MS/MS on a waters Q-Tof Ultima mass spectrometer at the Yale Cancer Center Mass spectroscopy and W.M. Keck Foundation and Biotechnology, followed by an automated Mascot algorithm against the NCBI database.

**Immunoprecipitation and Western blot analysis** 293T cells were transfected with plasmids as indicated and harvested 72 hr post transfection. The cells were lysed in WCEB lysis buffer. Lysates were cleared of cell debris by centrifugation. Antibodies (1–2 μg/mg protein) were added to the lysates and incubated at 4°C on a rotating device for 2 hr, and protein A agarose beads (Upstate, Temecula, CA) were added to precipitate the complex. Pelleted beads were washed and suspended in 4× SDS loading buffer, boiled and used for SDS-PAGE. The proteins were transferred onto the HyBond-P membrane (Amersham, UK). The membrane was probed with primary antibodies and the appropriate peroxidase-labeled secondary antibody, then visualized with an ECL system.

**CD44 minigene splicing assay** 293T cells were plated on 6-well plates and grown to 60-70% confluence and then transfected with the pCD44-v5 splicing reporter, pTip110-HA, pYB-1 or pYB-1 mutant plasmids. At 72 hr post transfection, total RNA was isolated using Trizol (Invitrogen, Carlsbad, CA), followed by acid:phenol extraction to prevent residual DNA from being used as a PCR template. Total RNA (0.6 μg) was used for reverse transcriptase (RT)-PCR using the Titan one tube RT-PCR system (Roche, Indianapolis, IN) and primers 5′-GAG GGA TCC GCT CCT GCC CC-3′ and 5′-CTC CCG GGC CAC CTC CAG TGC C-3′ and a program of 35 cycles of 94°C for 60s, 61°C for 60s, and 72°C for 90s. The RT-PCR products were separated on a 1% agarose gel.

**Data analysis** where appropriate, values were expressed as mean ± SD of triplicate experiments. All comparisons were made based on the control using two-tailed Student’s *t*-test. A *p* value of < 0.05 was considered statistically significant (*), *p* < 0.01 highly significant (**) and *p* < 0.001 strongly significant (***). All data were representative of multiple repeated experiments.

## Results

### Identification of Tip110-interacting proteins

**Figure 1:**
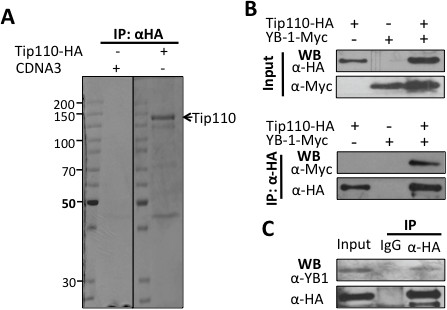
**Proteomic analysis of Tip110-binding cellular proteins including YB-1. A**. 293T cells were transfected with pTip110-HA. pcDNA3 was used in the mock transfection. Seventy-two hours post transfection, the cells were harvested for cell lysates. The lysates were applied to an anti-HA affinity matrix column, Tip110-binding proteins were then eluted from the column and analyzed on 10% SDS-PAGE followed by Coomassie blue staining. **B**. 293T cells were transfected with pTip110-HA, pYB-1-Myc or both and harvested 72 hr post transfection for cell lysates. pcDNA3 was added to equalize the total amount of transfected DNA. Cell lysates were directly used for Western blotting using anti-HA or anti-Myc antibody (top panels), or immunoprecipitated using anti-HA, followed by Western blotting using anti-HA or anti-Myc antibody (bottom panels). **C**. 293T cells were transfected with Tip110-HA and immunoprecipitation was performed using an anti-HA antibody or isotype-matched IgG, followed by Western blotting using anti-YB-1 or anti-HA antibody.

To identify Tip110-interacting protein, 293T cells were transfected with pTip110-HA plasmid. Cell lysates were prepared and passed through a HA-affinity column. Following extensive washes, the bound proteins were eluted and fractionated on the SDS-PAGE. In parallel, cell lysates from pcDNA3-transfected cells were also included. Coomassie blue staining revealed several protein bands whose intensity differed between samples from Tip110-HA and pcDNA3 (Figure 1A). Those proteins were recovered for mass spectrometric identification. Besides the bait protein Tip110 itself, there were 13 major proteins identified (Table 1). Those included cytoskeletal proteins, heat shock proteins, ribonucleoproteins, skin proteins and two ungrouped protein importin-2α and Y box binding protein 1 (YB-1). The interactions of Tip110 with all of those proteins were further examined and confirmed by immunoprecipitation followed by Western blot analysis (data not shown and see below). In the study, we chose to focus on YB-1 protein, as YB-1 and Tip110 appear to possess several similar functions. First, both Tip110 and YB-1 protein bind to RNA and are involved in post-transcriptional regulation such as pre-mRNA splicing [19,20]. Second, both Tip110 and YB-1 are highly expressed in some cancers [21]. Third, both Tip110 and YB-1 interact with HIV-1 Tat protein and regulate HIV-1 gene expression. Lastly, both Tip110 and YB-1 are regulated by transcription factor c-Myc [16,22]. Thus, all these observations imply that Tip110 forms complex with YB-1 and regulate each other’s function and suggest potential physiological significance of this interaction.

**Table 1:** Tip110-interacting proteins identified by IP followed by mass spectrometry

**Groups**	**Proteins**
Cytoskeletal Proteins	
	Actin
	Keratin-1/2
	Tubulin α/β
	Synaptagmin 2α
Heat Shock Proteins	
	HSP A8 (71 kDa)
	HSP A1B (70 kDa)
Ribonucleoproteins	
	hnRNPA2-B1
	hnRNP A1
	hnRNP U
Skin Proteins	
	Hornerin
	Filaggrin
Ungrouped Proteins	
	Importin-2α
	YB-1

The proteins were organized into groups based on their functions.

### Tip110 interacted with YB-1 and its molecular determinants

To confirm Tip110 interaction with YB-1, 293T cells were transfected with pTip110-HA, pYB-1-Myc, or both. Western blot analysis using anti-HA or anti-Myc antibody showed that both Tip110 and YB-1 were expressed in 293T cells (Figure 1B, top panels). Immunoprecipitation of cell lyastes using anti-HA antibody, followed by Western blotting with anti-Myc or anti-HA antibody showed that YB-1 was detected in the Tip110-immunoprecipitates only when Tip110-HA and Myc-YB-1 were co-expressed (Figure 1B, bottom panels). Immunoprecipitation and Western blotting were performed with the very same antibody to ensure the immnoprecipitation efficiency of the antibody. Similar experiments were also performed with 293T cells transfected with pTip110-HA only. Endogenous YB-1 was also detected in the Tip110-immunoprecipitates of Tip110-HA expression cells, but not present with the isotype IgG-immunoprecipitates (Figure 1C). Those results suggest that Tip110 complexed with YB-1 *in vivo*.

**Figure 2:**
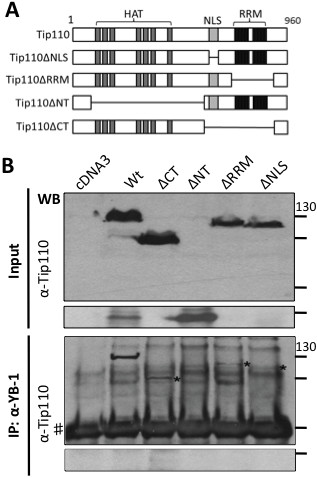
**Requirement of Tip110 domains for its binding to YB-1. A**. Scheme of Tip110 and its deletion mutants used in this study. HAT: half-a-tetratricopeptide repeat; NLS: nuclear localization signal; RRM: RNA recognition motif. **B**. 293T cells were transfected with pTip110-HA or each of Tip110 mutants. Cell lysates were directly used for Western blotting using anti-Tip110 antibody (top and middle panels), or immunoprecipitated using anti-YB-1 antibody, followed by Western blotting using anti-Tip110 (bottom panels). *: Expected proteins. #: Reactive IgG.

**Figure 3:**
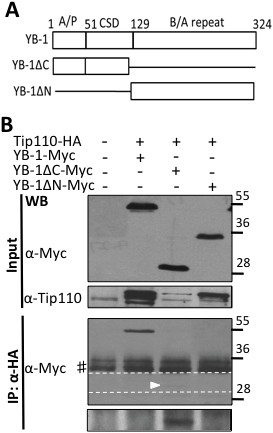
**Requirement of YB-1 domains for its binding to Tip110. A**. Scheme of YB-1 and its deletion mutants used in this study. A/P: Alanine/Proline rich; CSD: cold chock domain; B/A: basic/acidic repeats. **B**. 293T cells were transfected with pTip110-HA and pYB-1-Myc, or one of YB-1 mutants. Cell lysates were directly used for Western blotting using anti-Myc (top panel) or anti-Tip110 antibody (middle panel), or immunoprecipitated using anti-HA antibody, followed by Western blotting using anti-Myc (bottom panel). The long exposure of the area between the dotted lines was shown at the very bottom panel for the band marked by an arrowhead. #: Reactive IgG.

To further determine the specificity of the interaction, we took advantage of a series of Tip110 mutants that contained deletions of the N-terminal HAT-domain (ΔNT), RRM domain (ΔRRM), NLS domain (ΔNLS), and the C-terminal domain (ΔCT) (Figure 2A) [14]. 293T cells were transfected with pTip110-HA or each of the Tip110 mutants. Tip110 and its mutants were expressed at the expected molecular weights (Figure 2B, top two panels). Immunoprecipitation with anti-YB-1 antibody, followed by Western blot analysis using anti-Tip110 antibody showed detection of Tip110, ΔCT, ΔRRM and ΔNLS, but not ΔNT in the YB-1 immunoprecipitates (Figure 2B, bottom two panels). Similarly, we performed binding experiments of Tip110 to deletion mutants of YB-1 lacking the N-terminal (aa1-129, YB-1ΔN) or the C-terminal domains (aa130-324, YB-1ΔC) (Figure 3A) and found that deletion of the N-terminal domain of YB-1 (YB-1ΔN) prevented its complex formation with Tip110 (Figure 3B). Taken together, these results further confirmed the complex formation between Tip110 and YB-1 (Table 1) and suggest that the N-terminal domains of both Tip110 and YB-1 are directly involved in the complex formation.

### YB-1 modulated Tip110/Tat-mediated transactivation of the HIV-1 LTR promoter

**Figure 4:**
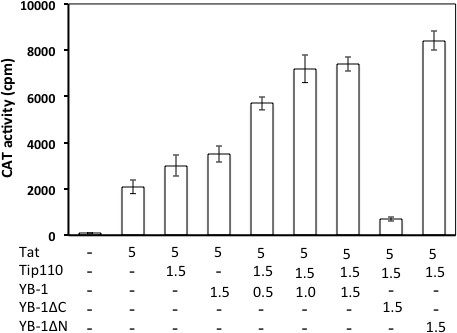
**Effects of YB-1 expression on Tip110/Tat-mediated transactivation of the HIV-1 LTR promoter.** 293T cells were transfected with 100 ng of pLTR-CAT and various combinations of pTat-Myc, pTip110-HA, pYB-1-Myc, or each of YB-1 mutants. Input amounts of plasmids were shown at the top, all were in micrograms (μg) with the except of pTat-Myc in nanograms (ng). pTat-Myc plasmid Cells were harvested 72 hr post transfection for the CAT reporter gene activity assay. pcDNA3 plasmid was added to equalize the total amount of DNA transfected, while pC3-GFP plasmid was used to ensure the transfection efficiency among transfections. The data were mean ± SD and representative of at least three independent experiments.

Tip110 interacts with the HIV-1 Tat viral protein and transactivates the HIV-1 LTR promoter [14,23]. Thus, we chose a LTR promoter-driven reporter gene chloramphenicol acetyltransferase (CAT) to determine the effects of YB-1 expression on this unique Tip110 function. Initial experiments were performed to optimize the input amounts of LTR-CAT, Tat, Tip110 and YB-1 expression plasmids to achieve a lower level of basal level LTR promoter activity and a modest level of Tat transactivation activity on the LTR promoter (data not shown). We then transfected 293T cells with LTR-CAT, Tat, Tip110 and/or increasing amounts of YB-1 expression plasmids. pcDNA3 was used to equalize the total amount of DNA transfected, while pC3-GFP was included in the transfections to normalize the transfection variations. As expected [14,23], Tip110 or YB-1 expression increased Tat-mediated transactivation of the LTR promoter activity (Figure 4). In the presence of Tip110, increased YB-1 expression led to further increase in the Tat-mediated LTR promoter activity. To further determine the relationship between the complex formation of Tip110 with YB-1 and YB-1 effects on Tip110/Tat-mediated transactivation of the LTR promoter, we performed similar experiments with both YB-1ΔN and YB-1ΔC mutants. YB-1ΔC mutant expression led to little CAT activity, while YB-1ΔN mutant showed similar CAT activity as the full-length YB-1. These results suggest that YB-1 expression enhanced Tip110/Tat-mediated transactivation of the LTR promoter and suggest that the complex formation of Tip110 and YB-1 is important for this function. The drastic reduction of the CAT activity in cells expressing YB-1ΔC to a background level suggests that YB-1ΔC may function in a dominant negative fashion.

### Tip110 promoted YB-1-mediated alternative splicing of CD44 minigene

**Figure 5:**
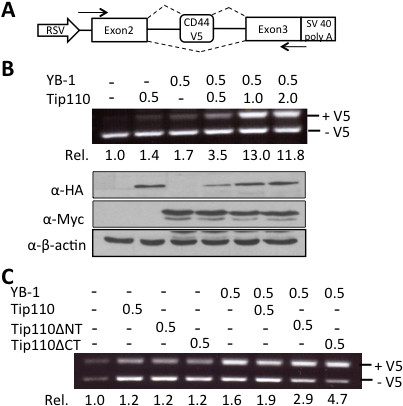
**Effects of Tip110 expression on YB-1-mediated alternative splicing of the CD44 minigene. A**. Schematic of the CD44 minigene containing the variable exon 5 (V5). The locations of the primer set used for RT-PCR were marked by arrows, the dotted lines indicated two alternative splicing possibilities: one with V5 (top, +V5), the other without V5 (bottom, -V5). **B**. 293T cells were transfected with the CD44 minigene plasmid, pYB-1-Myc, pTip110-HA, or both. Input amounts of plasmids were shown at the top, all were in micrograms (μg). Total RNA was isolated and used for RT-PCR. The ratio of V5 inclusion (+V5/–V5) was determined by densitometric scanning and the ImageJ software and was normalized to that transfected with the CD44 minigene only. Expression of YB-1 and Tip110 was determined by Western blotting. β-actin was the loading control. **C**. 293T cells were transfected with the CD44 minigene, pYB-1-Myc, pTip110-HA, or each of the Tip110 mutants. The V5 inclusion and quantitation were performed as stated above.

One of the well-characterized functions of YB-1 is regulation of the alternative splicing of the CD44 gene through interaction with the A/C-rich exon enhancer element [24]. Therefore, we next determined the effects of Tip110 on YB-1-mediated CD44 alternative splicing. We took advantage of a CD44 minigene (Figure 5A) and performed *in vivo* RT-PCR-based splicing assay. Initial experiments were performed to optimize the input amount of CD44 minigene, YB-1 and Tip110 expression plasmids to ensure the RT-PCR-based detection of the alternative splicing (data not shown). As expected [24], YB-1 expression led to increased inclusion of the variable exon 5 (V5) of the CD44 minigene (Figure 5B, top panels). Tip110 expression alone appeared to have the similar enhancement effects. In the presence of YB-1, Tip110 increased inclusion of the V5 exon of CD44 minigene in a dose-dependent manner. Tip110 and YB-1 expression were determined by Western blotting to ensure that Tip110 expression did not alter YB-1 stability (Figure 5B, bottom panels). We next determined the requirement of Tip110 binding to YB-1 for this function. Similar *in vivo* splicing assay was performed with Tip110ΔNT and Tip110ΔCT mutants. Compared to the full-length Tip110, Tip110ΔNT and Tip110ΔCT expression alone showed little changes in alternative splicing of the CD44 minigene (Figure 3C). Interestingly, in the presence of YB-1, Tip110ΔNT also showed little effects, while Tip110ΔCT had considerable increase in V5 inclusion of the CD44 minigene. These results suggest that Tip110 binding with YB-1 plays some roles in YB-1 function.

## Discussion

In this study, we took the immunoaffinity approach to enrich the Tip110-binding proteins and identified those proteins by mass spectrometry. This was followed by further characterization of Tip110 interaction with YB-1, YB-1 effects on Tip110-mediated transactivation of the HIV-1 LTR promoter and Tip110 effects on YB-1-mediated alternative splicing of CD44 gene. The results showed that Tip110 bound to YB-1 in a specific manner and the interaction mutually regulated each other’s function.

There were a total of 13 major cellular Tip110-binding proteins, which were grouped according to their function (Table 1). The group included cytoskeletal proteins, heat shock proteins, ribonucleoproteins, skin proteins and two ungrouped protein importin-2α and YB-1. Each of those proteins likely contributes to either known or unknown biological function of Tip110. For example, Tip110 binding to cytoskeletal proteins and skin proteins may be involved in a Tip110-related skin keratinization disorder called porokertosis. A mutation in Tip110 gene has been linked to this disease [25-27]. It is conceivable that Tip110 function in RNA metabolism including pre-mRNA splicing could require its interaction with ribonucleoproteins and heat shock proteins. The interaction of Tip110 with improtin-2α could also regulate Tip110 nuclear translocation in different cells and under various physiological conditions. The biological significance of all those interactions clearly warrants further investigation.

We focused on Tip110/YB-1 interaction in this study. YB-1 is a multi-functional protein and plays important roles in transcriptional and translational regulation, DNA repair, drug resistance and stress responses to extracellular signals (for review, see [28]). YB-1 has recently been shown to regulate pre-mRNA splicing [29]. Co-immunoprecipitation studies with tagged proteins and immunoprecipitation of endogenous and exogenous YB-1 with overexpressed Tip110 in 293T cells confirmed this protein-protein interaction (Figure 1B & C). Although Tip110 and YB-1 both contain RNA binding domains and have been shown to bind to RNA, the binding properties of Tip110 and YB-1 to each other were independent of RNA, as we showed that RNase A1 treatment in the cell lysates and during the immunoprecipitation did not alter their complex formation (data not shown). The specificity of the binding was further supported by the data obtained from mutagenesis analysis and showed that the N-terminal domains of both Tip110 and YB-1 were involved in the complex formation (Figures 2 and 3).

To investigate the functional relevance between the Tip110 and YB-1 interaction, we tested whether this complex formation affects transactivation of the HIV-1 LTR promoter and alternative splicing activity. Interaction of the HIV-1 Tat protein with its cognate RNA TAR is a prerequisite for Tat transactivation. Tip110 and YB-1 both interact with HIV-1 Tat viral protein and potentiate Tat transactivation in LTR-driven reporter gene assays [14,23]. Although YB-1 has only been shown to bind to the TAR region of the HIV-LTR, the synergistic effects of Tip110 are TAR-dependent, as these effects are attenuated by deletion of the TAR sequence from the HIV-1 LTR promoter. Based on these observations, we speculated that the Tip110/YB-1 complex affected the transactivation of HIV-1 LTR promoter activity. The LTR-CAT reporter gene assay showed that a fixed concentration of Tip110 combined with increasing concentrations of YB-1 resulted in a further increase in CAT activity (Figure 4), indicating that the Tip110/YB-1 complex modulates HIV-1 gene expression. The reporter gene assay with YB-1 mutants showed that expression of the YB-1ΔC mutant abolished CAT activity, while expression of the YB-1ΔN mutant enhanced the activation to a greater extent than the full length YB-1. One interesting observation we found during the course of this study was that expression of the YB-1ΔC mutant protein has a negative effect on Tip110 protein expression by Western blot (data not shown), which may explain the decrease in CAT activity. Furthermore, binding of Tat to YB-1 has been mapped to amino acids 75–203 in YB-1, while the YB-1ΔC mutant included amino acids 1–128. Therefore, there is a stretch of 85 amino acids (residues 128–203) that are important for Tat binding, that are deleted in the YB-1ΔC mutant, which may lead to the observed inhibition of CAT activity. Additional studies would be required to determine if the YB-1ΔC mutant could be utilized to impair Tat function and effect HIV-1 gene expression.

Alternative splicing represents an important nuclear mechanism in the post-transcriptional regulation of gene expression. The role of YB-1 protein in the alternative splicing of CD44 is well documented in the literature. YB-1 binds to A/C-rich exon enhancers and stimulates splicing of the CD44 alternative variable exon 4 [24]. CD44 is essential to the physiological activities of normal cells, but they are also associated with the pathologic activities of cancer cells (for review, see [30]). Pre-mRNA from the human CD44 gene undergoes extensive alternative splicing within a cassette of at least 10 exons [31]. Increasing inclusion of these exons has been correlated to cancer and metastasis [32,33]. Here, we utilized a CD44 minigene (Figure 5A) to determine the physiological function of the Tip110/YB-1 interaction. Our results showed that overexpression of YB-1 and Tip110 together in 293T cells enhanced the inclusion of the variable exon 5 from the CD44 minigene (Figure 5B). Furthermore, the N-terminal domain of Tip110 (pTip110ΔCT), which is involved in the interaction with YB-1, had higher alternative splicing activity than the C-terminal domain of Tip110 (pTip110ΔNT). These results demonstrate the physiological significance of Tip110/YB-1 complex formation on the alternative splicing regulation of CD44.

## Conclusions

There are a total of 13 cellular proteins that was identified by immunoaffinity purification followed by mass spectrometry. Among those is YB-1. Complex formation between Tip110 and YB-1 was confirmed by immunoprecipitation and Western blotting. Both N termini of Tip110 and YB-1 were found to be required for this complex formation. YB-1 expression enhanced Tip110-mediated transactivation of HIV-1 LTR promoter, and Tip110 expression increased YB-1-mediated CD44 pre-mRNA alternate splicing.

## Competing interests

The authors declare that they have no competing interests.

## Authors’ contributions

KT carried out the molecular genetic studies, participated in study design, and drafted the manuscript. YL participated in the study design and revised the manuscript. JJH conceived of the study, and participated in its design and coordination and helped to finalize the manuscript. All authors read and approved the final manuscript.
